# Virtually simulated interpersonal touch negatively affects perceived closeness and social affiliation to an avatar partner

**DOI:** 10.1038/s41598-024-51773-6

**Published:** 2024-01-16

**Authors:** Garima Saini, Maham Zain, Marigrace Noronha, Robert P. Bonin, Anna M. Lomanowska

**Affiliations:** 1 Department of Psychology, University of Toronto Mississauga, Mississauga, ON L5L 1C6 Canada; 2 Leslie Dan Faculty of Pharmacy, University of Toronto, Toronto, ON M5S 3M2 Canada; 3 University of Toronto Centre for the Study of Pain, University of Toronto, Toronto, ON M5S 3M2 Canada; 4 Transitional Pain Service, Department of Anesthesia and Pain Management, Toronto General Hospital, University Health Network, 200 Elizabeth St., Toronto, ON M5G 2C4 Canada

**Keywords:** Human behaviour, Sensory processing, Social behaviour

## Abstract

Interpersonal touch is an essential component of human non-verbal communication, facilitating social affiliation and bonding. With the widespread use of digital interfaces and online platforms in all realms of human interactions, there are fewer opportunities for communicating through touch. Popular online platforms that virtually simulate human interactions rely primarily on visual and auditory modalities, providing limited or no capacity for the exchange of tactile cues. Previous studies of virtual interactions have explored the simulation of social touch using haptic devices, but little is known about how the visual representation of interpersonal touch is perceived and integrated into a virtual social experience. In two studies we examined how the exchange of virtual touch mediated by simulated 3-dimensional human characters, or avatars, within an online virtual environment influenced affiliation towards an unfamiliar interaction partner. Surprisingly, the exchange of virtual touch negatively affected the perceived closeness and affiliation to the partner and the social evaluation of the interaction but did not affect the level of physiological arousal during the interaction. These results indicate that the visual representation of social touch is sufficient to virtually communicate touch-related cues that impact social affiliation, but the influence of touch may be dependent on the interaction context.

## Introduction

Interpersonal touch is frequently used as part of a repertoire of human non-verbal behaviours to communicate social meaning^1–3^ and plays an essential role in establishing and maintaining social bonds^4–7^. Touch is unique as a modality of non-verbal communication in that it requires close physical proximity between individuals to transmit information^3^. Even brief instances of interpersonal touch can influence social affiliation and prosocial behavior^8,9^, including positive social evaluation and attraction^10–12^, helping^13^, participation^14^, and compliance^15,16^. Interpersonal touch is also an inherently multimodal communication signal^1,17^. The perception of the touch stimulus, and even its somatosensory processing^18^, is modulated by relevant visual, auditory, and other cues about the socioemotional context of the touch and the identity of the toucher^17,19–21^. This multimodal nature of the touch experience is of particular interest at a time when innovations in social and virtual technologies^22^, alongside increased demand for remote and hybrid connectivity^23,24^, are propelling human interactions into the digital realm and limiting the exchange of touch^25–27^. Reduced opportunities for interpersonal touch^26^ became especially concerning during the COVID-19 pandemic that triggered widespread social distancing practices^28–30^ and an ensuing increase in online interactions^31^.

Advancements in digital technologies have included the development of haptic devices that can be used to produce vibro-tactile stimulation of the skin to simulate touch sensation^22,32^. However, haptic devices have yet to be widely incorporated into the digital platforms that are commonly used for online interactions. The primary means of exchanging interpersonal touch through digital interfaces at present is through visual representations, from simple features such as hug emoji or gifs^33,34^ to realistic simulation of touch gestures and even intimate interactions within online social virtual worlds and games^35,36^. Social virtual worlds (e.g., Second Life, IMVU, The Sims) in particular enable an immersive interaction experience where users can socialize in a virtual environment in the form of an avatar^37^, a 3D animated virtual character that represents the user^37,38^. Users tend to position their avatars in relation to each other in ways that align with typical social norms of the real world, with physical proximity and contact a common occurrence between pairs and groups of avatars interacting together^36,39,40^. Users often describe feeling like their avatar represents their own body and that the actions of the avatar are their own actions^38,41^. While the tactile component of physical contact between avatars is absent, visual components of these interactions are readily depicted on the screen^36^. Given the multimodal nature of social touch, an outstanding question is whether virtually simulating touch through the visual modality can serve as an effective means to communicate its social function.

Studies of multi-sensory integration in the experience of touch demonstrate that the manipulation of visual sensory input can give rise to the perception of touch^21^, with visual cues playing a dominant role in body perception^42,43^. A well-studies phenomenon referred to as the rubber hand illusion^42,44^ produces a sense of physical ownership of a rubber hand. This illusion can also be produced in various virtual reality (VR) set-ups^45–47^, with participants reporting feeling touch when viewing their virtual hand being touched by a virtual object. The illusory ownership of a virtual hand can also extend to the whole virtual body^48–50^. Furthermore, the intensity and pleasantness of virtual touch can be perceived differently depending on the type of virtual touch stimuli observed^51^, as well as who delivers the touch and to which part of the body^52^. Virtual touch stimuli can also be distinguished by different physiological responses as measured by skin conductance^51,52^. These findings demonstrate that the perception of touch and even its quality can be simulated through virtual interfaces; however, less is known about the social function of visualizing interpersonal touch in a virtual setting.

Classic studies demonstrate that interpersonal touch can facilitate social affiliation between strangers^8^. In a scenario referred to as the Midas Touch, a brief touch received from another person during a causal interaction increases helping behaviour, compliance, as well as liking for the toucher and the social exchange that occurred^10–13,15^. This effect can also be observed when touch is mediated remotely, through vibrotactile stimulation applied to the skin, during an online interaction^53,54^. Here, we devised a scenario in a virtual social environment where interpersonal touch is exchanged between avatars without tactile input. To promote the ecological validity of the study, we customized an existing virtual environment within IMVU (IMVU.com), a publicly accessible online social virtual world. IMVU is a popular online chatroom-based virtual world composed of thousands of virtual rooms generated by users, where users typically interact with each other via human-like avatars. A previous study conducted within the public IMVU platform demonstrated that interpersonal touch between avatars was frequently observed, especially in the context of dancing^36^. We thus employed a partner dance interaction that involved virtual touch or no touch between participant and confederate avatars and then assessed social affiliation towards the confederate. We hypothesized that virtual touch experienced through the visual modality would promote social affiliation as indicated by (1) closer seating distance to the confederate avatar after the dance and (2) self-reported impressions of closeness and affiliation towards the person behind the confederate avatar. We also assessed the physiological response to the virtual touch experience by measuring skin conductance, heart rate, and heart rate variability. As remotely mediated social touch has been shown to increase physiological arousal^20,55^, we hypothesized that virtual touch during the partner dance would stimulate greater physiological arousal than no touch.

## Methods (Study 1)

All study procedures were approved by the University of Toronto Mississauga Ethics Review Committee (2017-026) and were conducted in accordance with the principles of the revised Helsinki Declaration. Informed consent was obtained from all participants.

### Participants

Sixty-two undergraduate students (40 females) who were at least 18 years of age and reported normal or corrected-to-normal vision and hearing were recruited to participate in the study. Participants were randomly assigned to either the virtual Touch or virtual No-Touch condition. All participants interacted with an opposite sex avatar. Participants identifying their sex as male were paired with avatars exhibiting female secondary sexual characteristics, and vice versa for participants identifying their sex as female. One male participant from the No-Touch condition was excluded from the study due to oppositional behavior during the experiment, for a final total of 61 study participants. An a priori power analysis with an alpha of 0.05 and power = 0.8 (G*Power version 3.1.9.7, Dusseldorf, DE) showed that the estimated minimum sample size to detect a medium-large effect size for a two-tailed independent samples t-test is N = 52. Therefore, the obtained sample size is sufficient to test the study hypothesis.

### Virtual environment and avatars

**Figure 1:**
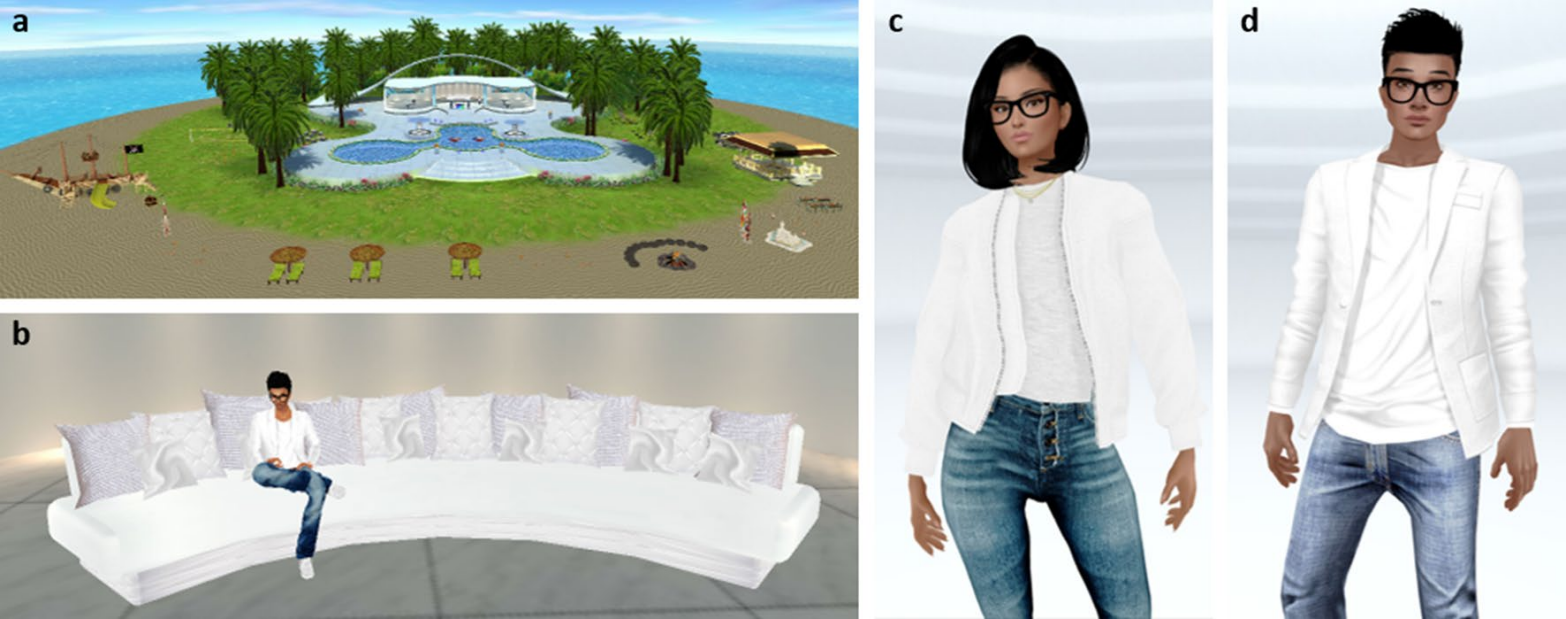
Representative still images of the virtual graphics seen by participants. (**a**) An aerial view of the virtual environment. (**b**) The couch scene used to measure interpersonal distance. Female (**c**) and male (**d**) confederate avatars used to interact with participants’ avatars.

**Figure 2:**
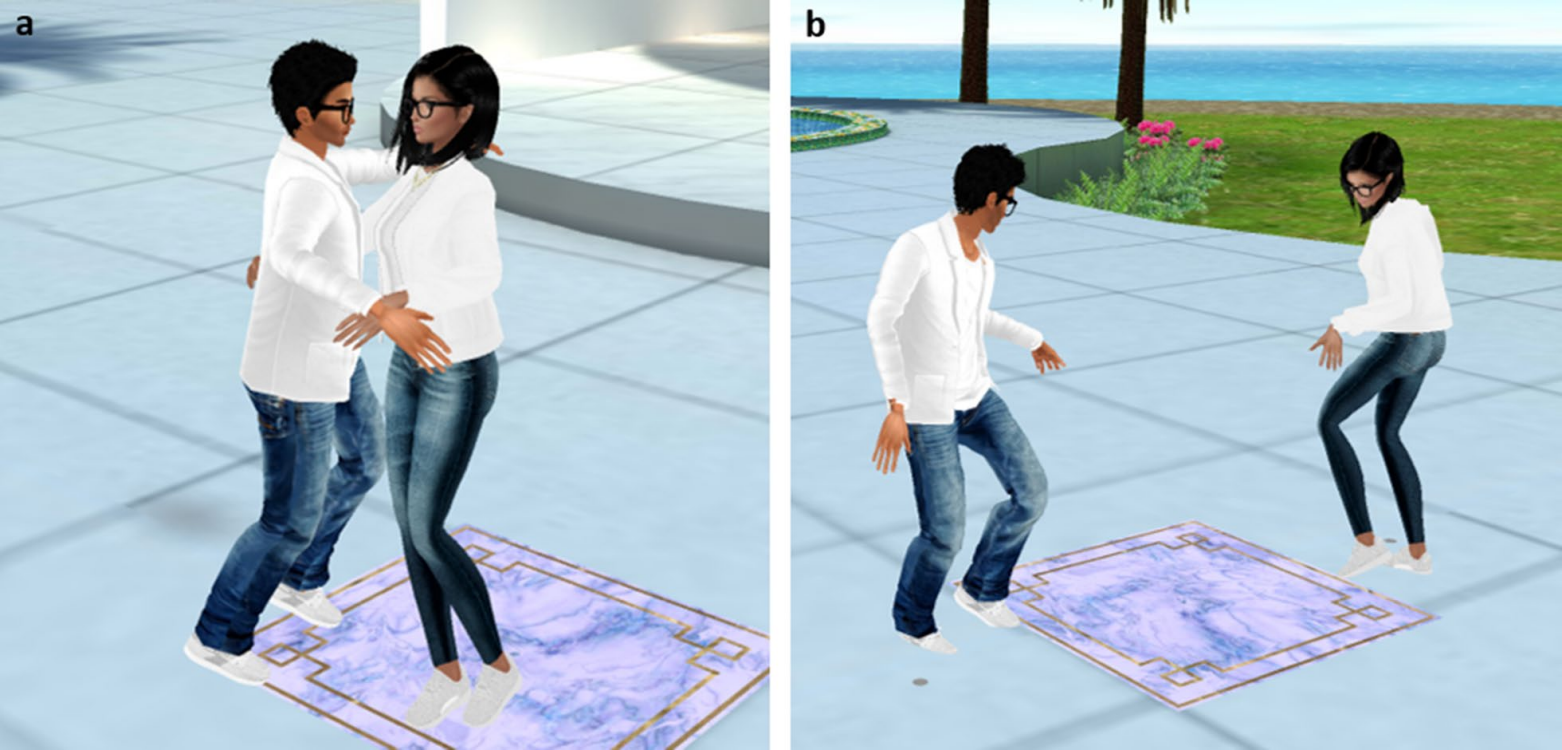
Still images of avatar dancing animations. Representative avatar positioning in the Touch (**a**) and No Touch (**b**) conditions during the virtual dance interaction. Pictured here are the male and female confederate avatars used in both studies.

The virtual environment was a private virtual chat room within the online IMVU platform (IMVU.com). The environment represented a tropical island surrounded by water, with a central swimming pool and pool deck area, a large sitting and dancing area with a disc-jockey booth, a beach area, and palm trees dispersed around the island (Fig. 1a). The environment also contained areas on which participants could click to place their avatar in a pose (e.g., sitting, standing) or an animation (e.g., playing a game of limbo, picking up seashells, yoga movements). The animations included three standard salsa style partner dances available in the IMVU software and appropriate for a general audience. The dances were portrayed through a series of changes in avatar body position in a repeating loop with instrumental salsa music playing in the background (see Supplementary Videos S1 and S2). In the Virtual Touch condition, dance animations involved the exchange of physical contact between avatars, including touching of the hands, torsos, or legs (Fig. 2a). In the Virtual No-Touch condition, there was no physical contact between avatars and instead avatars danced facing each other (Fig. 2b).

Each participant interacted with an opposite-sex confederate avatar (Fig. 1c,d). Two confederate avatars were selected from among standard avatars available within the IMVU platform and their appearance was styled using available customization features. The avatars’ clothing fully covered their torso, arms, and legs. The physical appearance of the male and female confederate avatars was matched as closely as possible in terms of clothing, hair color, skin color, and accessories. Avatars in the virtual environment could interact by typing in a chat box at the bottom of the screen, after which corresponding chat bubbles appeared above the avatars’ heads.

### Materials and procedure

The experiment took place in a window-less room, where a computer monitor connected to a PC computer was installed on a desk with a web camera placed on top of the monitor approximately at eye level with the participant. Participants were invited to sit on a chair in front of the monitor, were introduced to the study procedures, and provided written consent to participate. They were then asked to wear a wristband device on their non-dominant hand (E4 wristband, Empatica Inc.) that collected physiological measures during the experiment. The experimenter made sure not to touch the participant in any way while the participants settled into the experimental space and put on the wristband device. With their permission, participants were observed via webcam and remote screen sharing software (TeamViewer) during the experiment to ensure that they were paying attention to the screen and in case they encountered technical problems while they were participating in the experiment.

**Figure 3:**
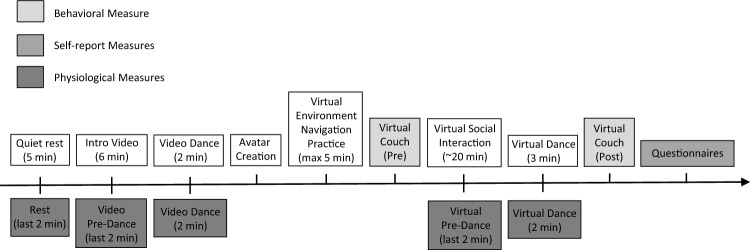
Experiment timeline for Study 1.

A diagram of the sequence of procedures is presented in Fig. 3. At the start of the experiment, participants were asked to remain seated and relax for 5 min alone in the experiment room to acclimate to the setting. Next, the experimenter returned to the room and provided participants with a paper invitation listing an itinerary of activities to be completed in the virtual environment, including virtual salsa dance lessons at the end. The experimenter then left the room and participants watched a 6 min introductory video providing an overview of the procedures involved in creating an avatar, moving about in the virtual world, and completing activities around the virtual island while interacting with another avatar. The last 2 min of the introductory video demonstrated two avatars engaging in the three dance animations specific to the participant’s experimental condition (Virtual Touch or No Touch; see Supplementary Videos S1 and S2).

Next, the experimenter entered the room to further instruct participants on what will happen during the session, including how to access IMVU, create an avatar, and proceed with the activities on the virtual island. Participants were given a temporary email address to activate their IMVU account and instructions to create their own avatar, after which participants were again left alone in the room. The participants were instructed to create their avatar in a way that the avatar resembled them physically as much as possible. Next, participants entered a practice virtual environment, a standard penthouse apartment provided by IMVU, to acclimate to navigating the environment and the avatar. The practice session lasted up to a maximum of 5 min. Finally, participants received a digital invite to enter the virtual island. The instructions indicated that when they enter the virtual island, participants will meet a second avatar who is another participant completing the experiment simultaneously in a different room. Participants were instructed to interact with the second avatar by typing in the chat. The second avatar was controlled by a confederate research assistant who operated the avatar from another computer in a separate room.

Once participants entered the virtual island environment, the confederate avatar started to chat by typing “Hello” and asking the participant to join them in sitting on the virtual couch. The couch had 6 seating locations and the confederate avatar was already seated on the couch at the second position from the left, leaving the other 5 spots open for the participant’s avatar (Fig. 1b). The confederate avatar continued to chat with the participant as they completed the activities on the virtual island. The activities included exploring the pool area, playing volleyball and limbo, exploring a pirate ship and treasure chest, driving a boat on water, picking up seashells and building a sandcastle at the beach, and yoga practice.

The confederate had a script of chat prompts to maintain a consistent flow of conversation across participants. The prompts focused on the activities on the virtual island. The confederate responded to the participant’s chat messages in a friendly way and steered away from personal topics. The chat exchanges were recorded and quantified to ensure that the chat characteristics were consistent across study conditions.

For the last “dance lesson” activity, participants danced with the confederate to three salsa routines, with each dance routine lasting approximately 1 min. Following the dance activity, the confederate returned to sit on the virtual couch and asked the participant to join them. The confederate avatar sat at the second position from the left, in the same way as at the start of the virtual interaction. The virtual experience on the island ranged from 20 to 25 min on average. The experimenter entered the room once during this time to briefly check on the participant and to also reinforce the impression that they were not the ones controlling the other avatar. When the virtual interaction ended, participants were asked to complete questionnaires provided on the same computer.

### Measures

#### Interpersonal distance

Interpersonal distance refers to the physical space between individuals and is considered as a component of non-verbal communication^56,57^. It can be used as a behavioral indicator of social affiliation, where closer proximity typically signals affiliation and greater distance signals avoidance or exclusion^58–60^. Studies in virtual settings demonstrate that interpersonal distance is a relevant factor in how individuals position their avatars in relation to other avatars within the same virtual representation of physical space^39,61,62^. The distance between avatars interacting in virtual settings affects the behavioural, self-report, and physiological responses to the interaction^57,63,64^. In the present study, interpersonal distance was used as a behavioral readout of the influence of virtual touch on social affiliation. We recorded how far participants positioned their avatar in relation to the confederate avatar on the virtual 6-seater couch at the start and the end of the virtual interaction. Recorded measures included the number of sitting spaces (range 0–3) the participant avatar left between themselves and the confederate avatar at the start and the end of the virtual interaction, as well as the difference in sitting spaces between these two time points.

#### Self-report measures

Participants completed self-report measures in the following order: Inclusion of Other in the Self scale, Interpersonal Impressions questionnaire, previous experience, and demographic questions. Qualtrics software (www.qualtrics.com) was used to administer the questions.

##### Demographics and previous experience

In the demographic portion of the questionnaire, participants were asked to report their sex and age. Participants also answered questions regarding their prior experience in virtual worlds, including whether they previously played in an online virtual environment (Yes/No) and how often (5-point scale from “Never” to “Daily”), and whether they have ever played in IMVU and how often. The results were divided into two categories representing little virtual experience (“Never” or “Once per year”) or frequent virtual experience (“Once per month,” “Weekly,” or “Daily”).

##### Perceived closeness

The Inclusion of Other in the Self scale (IOS)^65^ was used to measure participants’ perception of closeness towards the person behind the other avatar. Using the online continuous version of the IOS scale^66^, participants were asked to respond to the following prompt “Please use the diagram below to describe how you currently feel towards the person behind the other avatar.” Participants responded by moving a sliding cursor on a computer screen to adjust the position of two circles in relation to each other on a horizontal axis. The left circle was identified on the screen with the word “Self” and the right circle with the word “Other.” In the starting position, the circles were farthest from each other (two circle widths apart, recorded as 0 arbitrary units). The closest possible position allowed for the circles to overlap completely (recorded as 300 arbitrary units). The measure of *Perceived Closeness* was the distance participants moved the cursor to bring the circles together, with higher values indicating greater closeness.

##### Interpersonal impressions

Participants’ *Interpersonal Impressions* were measured with 12 questions in two categories. The first category included five questions related to the *experience* of interacting with the person controlling the other avatar (“I felt like I was close to the other person during our interactions”, “I feel like I got to know the other person well”, “I liked exploring the island with the other person”, “I liked having a conversation with the other person”, “I liked dancing with the other person”). The second category included seven questions adapted from^67^ regarding *affiliation* towards the person controlling the other avatar (e.g., “I like the other person a lot”, “I feel I could talk to the other person easily”, “I feel comfortable with the other person”, “I would enjoy interacting with the other person again”, “If I had the opportunity, I would like to meet the other person in real life”, “I feel I could trust the other person with a secret”, “I am friends with the other person”). Questions were rated on a 7-point scale from “strongly agree” to “strongly disagree”.

#### Physiological measures

A wristband device (Empatica E4) was used to collect and report information on electrodermal activity (EDA), heart rate (HR) and heart rate variability (HRV) throughout the experiments. Physiological measures were collected in the same way in both Study 1 and 2 and data from both studies were combined due to the loss of some data that resulted in reduced sample sizes. The procedures are described in further detail in Study 2.

### Statistical analysis

Independent sample t-tests were used to compare the mean scores between participants in the Touch and No Touch condition on the Inclusion of Other in Self scale, Interpersonal Impressions questionnaire, and participant age. Chi-squared tests were used to compare the numbers of male and female participants and prior experience in virtual worlds across the two study conditions. An independent samples non-parametric Mann–Whitney U test was used to compare the seating distance between avatars across the two study conditions. A Wilcoxon signed-rank test was used to compare the seating distance before and after the virtual experience in each study condition.

Statistical analyses were conducted using SPSS 28.0 (IBM Corp., Somers, NY) with the rejection level set at p < 0.05. All data are presented in figures as means ± SEM, unless otherwise indicated.

## Results (Study 1)

### Demographics and previous experience

Demographic and previous experience results are presented in Table 1. A t-test analysis revealed no significant difference across the Touch and No-Touch conditions in participant age. Chi square analyses revealed that participants in the two conditions also did not differ in the number of those reporting male and female sex or in prior experience in online virtual worlds. Only 4 participants (2 in each condition) had prior experience using IMVU.

**Table 1:** Participant demographics and prior experience in virtual worlds.

	Variable	Touch	No touch	*P*
Study 1	n	32	29	
	Age (years)	19.9 (2.7)^a^	19.9 (1.7)	0.987
	Sex (female)	21	19	0.993
	Experienced in virtual worlds^b^	11	11	0.773
﻿Study 2	n	19	19	
	Age (years)	20.2 (2.7)^a^	20.8 (2.9)	0.489
	Sex (female)	11	11	1.000
	Experienced in virtual worlds^b^	7	5	0.414

^a^Mean (SD).

^b^Reported as “Once per month,” “Weekly,” or “Daily”.

### Chat exchanges

The chat exchanges between each participant and the confederate were quantified to examine how they compared across study conditions. T-test comparisons showed that there were no significant differences between the Touch and No Touch conditions in the number of messages sent by the confederate (Touch: *M* = 76.5, *SD* = 15.5; No Touch: *M* = 79.9, *SD* = 14.4; *t*(59) = 0.87, *p* = 0.389, *d* = 0.23) and the participants (Touch: *M* = 52.9, *SD* = 21.1; No Touch: *M* = 54.1, *SD* = 17.5; *t*(59) = 0.25, *p* = 0.802, *d* = 0.06) or in the total message word count for the confederate (Touch: *M* = 417.3, *SD* = 117.8; No Touch: *M* = 422.3, *SD* = 70.3; *t*(58) = 0.20, *p* = 0.844, *d* = 0.05) and the participants (Touch: *M* = 210.2, *SD* = 133.9; No Touch: *M* = 195.4, *SD* = 93.7; *t*(58) = 0.49, *p* = 0.624, *d* = 0.13).

### Interpersonal distance

The seating distance (Table 2) between the participant and confederate avatar before and after the virtual interaction was used as a measure of interpersonal distance. Independent samples non-parametric Mann–Whitney U tests revealed that there were no significant differences between the Touch (start: *Mdn* = 0; end: *Mdn* = 1) and No Touch (start: *Mdn* = 0; end: *Mdn* = 1) conditions in seating distance at the start (*U* = 445, *p* = 0.961, *r* = 0.00), as well as at the end (*U* = 425, *p* = 0.700, *r* = *0.01*) of the virtual interaction. A related-samples Wilcoxon signed rank test revealed that there was no significant difference when comparing seating distance before and after the virtual interaction in both the Touch (*Z* = 1.16, *p* = 0.248, r = 0.20) and No Touch (*Z* = 1.11, *p* = 0.265, *r* = 0.20) conditions.

**Table 2:** Number of participants (n) in each seating distance configuration before and after the virtual interaction.

Condition	Seats between avatars (seating distance) ^a^	Study 1 (n)		Study 2 (n)	
		Before	After	Before	After
Touch	0	17	14	8	7
	1	8	9	3	3
	2	4	6	2	2
	3	2	2	0	1
No touch	0	15	11	4	8
	1	9	10	6	7
	2	2	6	7	3
	3	2	1	2	1

Only participants with both before and after measures are included (2 participants excluded in Study 1; 1 participant excluded in Study 2).

^a^Number of seats separating participant and confederate avatars.

### Perceived closeness

**Figure 4:**
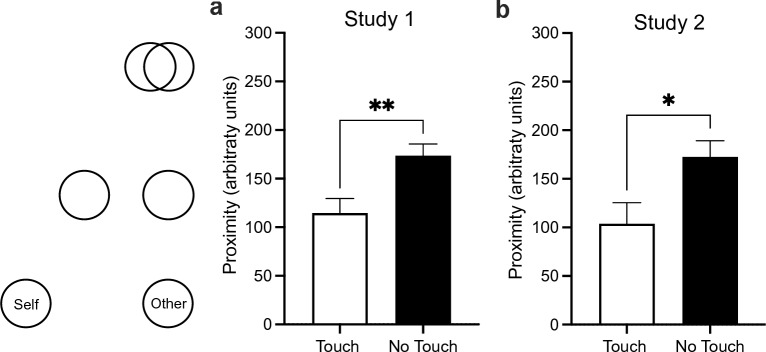
Reported closeness to the person behind the partner avatar on the Inclusion of the Other in the Self Scale (IOS). Increasing values (mean ± SEM) indicate closer proximity of the self to the other. In both Study 1 (**a**) and 2 (**b**), participants in the No Touch condition indicated significantly greater closeness towards the person behind the other avatar compared to participants in the Touch condition. *p < 0.05; **p < 0.01.

The IOS scale was used to measure the participants’ perceived interpersonal closeness to the partner avatar. An independent samples t-tests revealed that participants in the No Touch condition indicated significantly greater closeness towards the person behind the other avatar compared to participants in the Touch condition, *t*(58) = 3.02, *p* = 0.004, *d* = 0.78 (Fig. 4a).

### Interpersonal impressions

**Figure 5:**
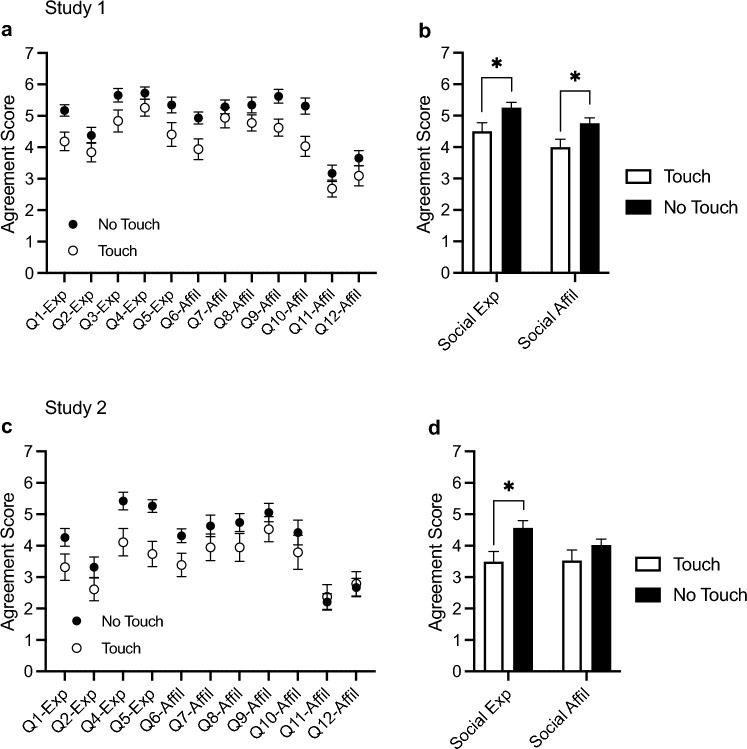
Responses on the Interpersonal Impressions questionnaire. (**a**, **c**) Mean (± SEM) agreement scores on each of the Interpersonal Experience (Q1–5) and Interpersonal Affiliation (Q6–12) questions in Study 1 and 2. (**b**, **d**) Pooled mean (± SEM) agreement scores on questions in the Interpersonal Experience and Affiliation categories in Study 1 and 2. Participants in the No Touch condition reported a significantly more positive impression of their interpersonal experience in both Study 1 and 2, and significantly greater affiliation towards the person behind the other avatar in Study 1. *p < 0.05.

Participants’ impressions of the virtual interaction experience and the partner avatar were assessed using 12 Interpersonal Impressions questions. Figure 5a shows the average responses of the participants to each of the questions and Fig. 5b shows the pooled average responses for questions in the interpersonal *experience* (questions 1–5) and *affiliation* (questions 6–12) categories. An independent samples t-tests revealed that participants in the No Touch condition reported a significantly more positive impression of their interpersonal *experience, t*(59) = 2.28, *p* = 0.027, *d* = 0.58, and *affiliation* towards the person behind the other avatar, *t*(59) = 2.45, *p* = 0.017, *d* = 0.63.

### Physiological responses

Physiological responses of participants were assessed throughout the experiment. Electrodermal and cardiac data from Study 1 and Study 2 were collapsed together to account for the low sample size of collected data in each study. The results are presented together in Study 2.

### Exploratory analyses of sex differences

Information about participants’ sex was collected for pairing with opposite-sex avatars. We conducted exploratory analyses to assess whether participants’ reported sex had any effects on perceived closeness (IOS scale) and interpersonal impressions (interpersonal *affiliation* and *experience*) of the partner avatar. Two-way ANOVAs with the factors of condition (Touch, No Touch) and sex (Female, Male) revealed no significant main effects of sex and no interactions with the experimental condition in any of these measures (see Supplementary Tables S1 and S2 for details).

## Discussion (Study 1)

The results of Study 1 demonstrate that the experience of exchanging interpersonal virtual touch with another avatar through the visual modality reduces self-reported social affiliation, including perceived closeness and impressions, towards the person behind the other avatar. This finding does not support our hypothesis that virtual touch would enhance social affiliation, as is typically observed in face-to-face interactions^10–12^ and when interpersonal touch is mediated remotely via a haptic device^53,54^. To confirm these findings, we conducted a replication study in a new cohort of participants (Study 2) and focused more directly on the virtual touch interaction itself. In Study 2, participants were invited to proceed to the virtual dance lesson with the confederate avatar directly after meeting them in the virtual environment. As in Study 1, participants were assigned to a Touch or a No Touch condition.

## Methods (Study 2)

All study procedures were approved by the University of Toronto Mississauga Ethics Review Committee (2017-026) and were conducted in accordance with the principles of the revised Helsinki Declaration. Informed consent was obtained from all participants.

### Participants

Based on the medium-large effect sizes from the self-report measures obtained in Study 1, we estimated that approximately 40 participants would be sufficient to detect a medium-large effect size for a two-tailed independent samples t-test with an alpha = 0.05 and power = 0.8 (G*Power version 3.1.9.7, Dusseldorf, DE). Thirty-nine undergraduate students (23 females) who were at least 18 years of age and reported normal or corrected-to-normal vision and hearing were recruited to participate in the study. As in the first study, participants were randomly assigned to either the virtual Touch or virtual No-Touch condition and all participants interacted with an opposite sex avatar. One female participant from the Touch condition did not complete the experiment due to a fire alarm, for a final total of 38 study participants.

### Materials and procedure

The materials and procedures were the same as in Study 1, except for the following modifications: instead of engaging in 20 min of activities on the virtual island with the confederate avatar, participants only engaged in the salsa dance lessons. The virtual island in Study 2 also featured a disc-jockey (DJ) character who danced along with the music at the DJ booth and instructed the participant and confederate avatars via chat messages when to sit on the virtual couch and when to start and stop the dance lessons. The virtual experience on the island lasted about 10 min in total.

The confederate avatar maintained a minimum amount of chat with the participant by exchanging pleasantries according to a pre-determined script. The chat exchanges were recorded and quantified to ensure that the chat characteristics were consistent across study conditions.

### Measures

#### Interpersonal distance

The seating distance between each participant’s avatar in relation to the confederate avatar on the virtual 6-seater couch at the start and the end of the virtual interaction was recorded in the same way as in Study 1.

#### Self-report measures

At the end of the virtual interaction, participants were asked to complete the same self-report measures as in Study 1.

#### Physiological measures

A wristband device (Empatica E4) was used to collect and report information on electrodermal activity (EDA), heart rate (HR) and heart rate variability (HRV) throughout the experiments. Recordings from the hotoplethysmography were sampled at 64 Hz and used in the estimation of HR and HRV. EDA data was obtained from the electrodermal activity sensor and sampled at 4 Hz. The Empatica E4 has been shown to produce reliable and consistent HR and HRV data when compared to other validated tools^68,69^. While the device does result in significantly different absolute values for EDA measures, this has been shown to result from recording at the wrist as opposed to the fingers^70,71^. Skin conductance data obtained from this device has yielded high stress discrimination power^71^. Participants put the device on themselves to avoid physical contact with the experimenter. They were instructed to wear the device on their non-dominant hand with the electrodes positioned on the inside of the wrist, lined up in parallel with the ring finger. Participants were asked to remove any jewelry, watch, or wristband worn on that wrist.

All data from the wristband device were extracted through the Empatica Connect online platform (https://www.empatica.com/connect/). Data for five continuous time periods (events) were extracted (see Fig. 3): the final 2 min of the initial rest period (Rest), 2 min while watching the introduction video of the virtual environment (Video Pre-Dance), 2 min while watching the virtual dance on the introductory video (Video Dance), 2 min prior to participating in the virtual dance for Study 1 and the final two min spent in the virtual penthouse for Study 2 (Virtual Pre-Dance), and the first 2 min while participating in the virtual dance (Virtual Dance).

EDA data were processed using the continuous decomposition analysis via the Ledalab extension for MATLab r2018a to separate the tonic and phasic components of the signal. Default deconvolution parameters were used. Physiological arousal during each 2 min event was measured as the mean tonic skin conductance level (SCL) and the frequency of skin conductance responses (SCR). The minimum signal amplitude for detecting SCRs was set at 0.05 μS.

Cardiac data were analyzed using Standard Kubios HRV software. The data were visually screened for artifacts and corrected for using the artifact correction tool in Kubios HRV with the threshold set at the lowest setting. Based on these parameters, any intervals deviating by more than 0.45 s from the average interbeat interval of that participant were marked as an artifact and removed. The average heart beats per minute (HR) and standard deviation of the interbeat intervals (SDNN) were used to assess heart rate and heart rate variability, respectively, during each 2 min event. SDNN was chosen instead of other commonly reported measures of HRV, such as the root mean squared differences of successive difference of intervals (RMSSD), as SDNN values collected from the Empatica E4 are more reliable and consistent with other validated sensors^69^.

### Statistical analysis

The statistical analyses for behavioral and self-report measures were the same as in Study 1. Data for Study 1 and Study 2 were analyzed separately, except for physiological measures.

Data for physiological measures from Study 1 and Study 2 were analyzed together due to the loss of some data leading to reduced sample sizes. Independent samples t-tests were used to compare measures of EDA and cardiac activity at the end of the initial rest period between participants assigned to the two conditions (Touch, No Touch). Three-way repeated measures ANOVAs were used to assess the change in EDA and cardiac activity across the within-subjects factors of *event* (Pre-Dance, Dance) and *media* (Video, Virtual), and the between-subjects factor of *condition* (Touch, No Touch). The same analyses were also conducted with data from each study treated separately and the results are included in Supplementary Tables S3–S6.

Statistical analyses were conducted using SPSS 28.0 (IBM Corp., Somers, NY) and GraphPad Prism 9.4.1 (San Diego, CA) with the rejection level set at p < 0.05. All data are presented in figures as means ± SEM, unless otherwise indicated.

## Results (Study 2)

### Demographics and previous experience

Demographic and experience results are presented in Table 1. A t-test analysis revealed no significant difference across the Touch and No-Touch conditions in participant age. Chi square analyses revealed that participants in the two conditions also did not differ in the number of those reporting male and female sex or in prior experience in online virtual worlds. No participants had prior experience using IMVU.

### Chat exchanges

The chat exchanges between each participant and the confederate were quantified to examine how they compared across study conditions. Two participants in the Touch condition did not engage in any chat with the confederate and their data were not included in this analysis. T-test comparisons showed that there were no significant differences between the Touch and No Touch conditions in the number of messages sent by the confederate (Touch: *M* = 21.1, *SD* = 4.3; No Touch: *M* = 23.1, *SD* = 3.3; *t*(34) = 1.58, *p* = 0.124, *d* = 0.52) and the participants (Touch: *M* = 17.5, *SD* = 6.1; No Touch: *M* = 19.5, *SD* = 4.4; *t*(34) = 1.15, *p* = 0.259, *d* = 0.34) or in the total message word count for the confederate (Touch: *M* = 95.5, *SD* = 20.0; No Touch: *M* = 103.6, *SD* = 15.2; *t*(34) = 1.38, *p* = 0.178, *d* = 0.46) and the participants (Touch: *M* = 78.2, *SD* = 33.8; No Touch: *M* = 85.0, *SD* = 33.0; *t*(34) = 0.61, *p* = 0.544, *d* = 0.20).

### Interpersonal distance

The seating distance (Table 2) between the participant and confederate avatar before and after the virtual interaction was again used as a measure of interpersonal distance. Independent samples non-parametric Mann–Whitney U tests revealed that there was a significant difference between the Touch (*Mdn* = 0) and No Touch (*Mdn* = 1) conditions in seating distance at the start (*U* = 66, *p* = 0.010, *r* = 0.45) of the virtual interaction, but no significant difference between the Touch (*Mdn* = 0) and No Touch (*Mdn* = 1) conditions at the end (*U* = 114, *p* = 0.695, *r* = 0.07). A related-samples Wilcoxon signed rank test revealed that there was no significant difference when comparing seating distance before and after the virtual interaction in both the Touch (*Z* = 0.59, *p* = 0.558, *r* = 0.16) and No Touch (*Z* = 1.73, *p* = 0.083, *r* = 0.40) conditions.

### Interpersonal closeness

The IOS was again used to measure the participants’ perceived interpersonal closeness to the partner avatar. Similarly to Study 1, an independent samples t-tests revealed that participants in the No Touch condition indicated significantly greater closeness towards the person behind the other avatar compared to participants in the Touch condition, *t*(36) = 2.50, *p* = 0.017, *d* = 0.81 (Fig. 4b).

### Interpersonal impressions

Participants’ impressions of the virtual interaction experience and the partner avatar were again assessed using 12 Interpersonal Impressions questions. Figure 5c shows the average responses of the participants to each of the questions and Fig. 5d shows the pooled average responses for questions in the interpersonal *experience* (questions 1–5) and *affiliation* (questions 6–12) categories. An independent samples t-tests revealed that participants in the No Touch condition reported a significantly more positive impression of their interpersonal *experience, t*(36) = 2.69, *p* = 0.011, *d* = 0.87, but the difference across conditions in interpersonal *affiliation* was not significant, *t*(36) = 1.28, *p* = 0.212, *d* = 0.41.

### Physiological responses

Participants’ physiological responses were measured using a wristband device (Empatica E4) throughout the experiment and data were analyzed for five continuous 2 min time periods (events; see Fig. 3). Electrodermal and cardiac data from Study 1 and Study 2 were analyzed together to account for the low sample size of available data in each study.

#### Electrodermal activity

**Figure 6:**
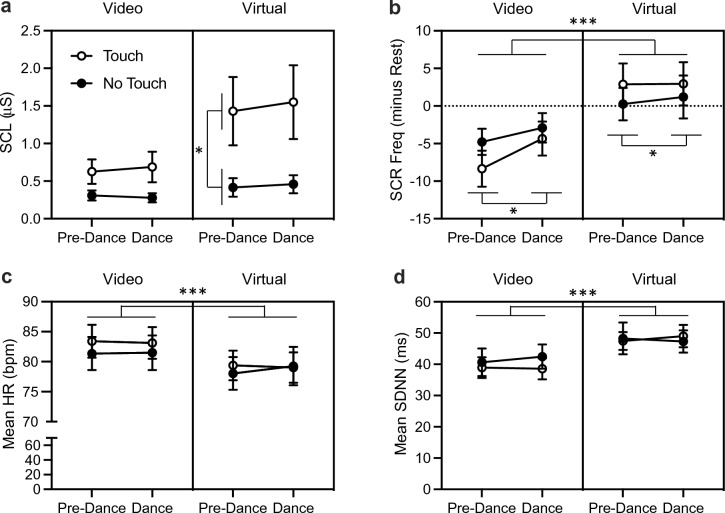
Physiological responses to Video and Virtual stimuli. (**a**) Participants in the Touch condition had significantly higher skin conductance level (SCL) values compared to the No Touch condition during virtual interactions. (**b**) Adjusted skin conductance responses (SCRs) were significantly more frequent during virtual interactions compared to video watching. The frequency of SCRs was also significantly higher during the Dance events compared to the Pre-Dance events. (**c**) Mean heart rate (HR) was significantly lower during virtual interactions compared to video watching. (**d**) Conversely, heart rate variability, measured by the mean standard deviation of the interbeat intervals (SDNN), was significantly higher during virtual interactions compared to video watching. All values represent mean (± SEM); *p < 0.05; ***p < 0.001.

After data extraction and processing, complete data were available from 41 participants in the Touch condition and 44 participants in the No Touch condition. An independent samples t-test on the absolute SCL values during the initial rest period revealed no significant differences between participants assigned to the Touch (*M* = 0.64, *SD* = 0.98) and No Touch condition (*M* = 0.36, *SD* = 0.54). A repeated measures three-way ANOVA revealed a significant main effect of *media*, *F*_(1, 83)_ = 8.75, *p* = 0.004, *η*_p_^2^ = 0.10, where the SCL was higher when the participants were actively interacting as avatars in the virtual environment compared to passively watching a video of avatars interacting in the same environment (Fig. 6a). There was also a significant main effect of *condition*, *F*_(1, 83)_ = 4.94, *p* < 0.029, *η*_p_^2^ = 0.06, where participants in the Touch condition had higher SCL levels overall than participants in the No Touch condition. There was also significant interaction between *media* and *condition*, *F*_(1, 83)_ = 4.36, *p* < 0.040, *η*_p_^2^ = 0.05. Follow-up simple effects analyses revealed that participants in the Touch condition had significantly higher SCL levels than participants in the No Touch condition when actively interacting in the virtual environment *F*_(1, 83)_ = 4.99, *p* < 0.028 *η*_p_^2^ = 0.06. However, there was no significant difference between conditions when participants were passively watching a video of avatars interacting *F*_(1, 83)_ = 3.77, *p* < 0.056, *η*_p_^2^ = 0.04. There were no other significant main effects or interactions. Results of the same analyses of SCL values conducted separately for Study 1 and Study 2 are available in Supplementary Table S3.

An independent samples t-test on the SCR frequency during the initial rest period revealed a significant difference between participants assigned to the Touch (*M* = 16.24, *SD* = 4.0) and No Touch condition (*M* = 6.89, *SD* = 2.07), *t*(83) = 2.136, *p* = 0.0356, *d* = 0.46. To account for this baseline difference, we subtracted the values obtained during the rest period from values obtained during the other events of interest. A repeated measures three-way ANOVA on the corrected values revealed a significant main effect of *media*, F_(1, 83)_ = 15.19, p < 0.001, *η*_p_^2^ = 0.15, where the frequency of SCRs was higher when the participants were actively interacting as avatars in the virtual environment compared to passively watching a video of avatars interacting in the same environment (Fig. 6b). There was also a significant main effect of *event*, F_(1, 83)_ = 6.69, p = 0.011, *η*_p_^2^ = 0.07, where the frequency of SCRs was higher during the Dance events compared to the Pre-Dance events. There were no other significant main effects or significant interactions. Results of the same analyses of SCR frequency conducted separately for Study 1 and Study 2 are available in Supplementary Table S4.

#### Cardiac activity

After data extraction and processing, complete data were available from 22 participants in the Touch condition and 20 participants in the No Touch condition. An independent samples t-test analysis of the absolute HR values during the initial rest period revealed no significant differences between participants assigned to the Touch (*M* = 85.37, *SD* = 13.73) and No Touch condition (*M* = 83.20, *SD* = 12.17). A repeated measures three-way ANOVA revealed a significant main effect of *media*, *F*_(1, 40)_ = 26.50, *p* < 0.0001, *η*_p_^2^ = 0.40, where mean HR was lower when the participants were actively interacting as avatars in the virtual environment compared to passively watching a video of avatars interacting in the same environment (Fig. 6c). There were no other significant effects or significant interactions. Results of the same analyses of HR values conducted separately for Study 1 and Study 2 are available in Supplementary Table S5.

An independent samples t-test analysis of the absolute SDNN values during the initial rest period revealed no significant differences between participants in the Touch (*M* = 46.73, *SD* = 20.59) and No Touch condition (*M* = 45.75, *SD* = 16.69). A repeated measures three-way ANOVA revealed a significant effect of *media*, *F*_(1, 40)_ = 16.38, *p* < 0.001, *η*_p_^2^ = 0.29, where SDNN values were higher when the participants were actively interacting as avatars in the virtual environment compared to passively watching a video of avatars interacting in the same environment (Fig. 6d). There were no other significant main effects or significant interactions. Results of the same analyses of SDNN values conducted separately for Study 1 and Study 2 are available in Supplementary Table S6.

### Exploratory analyses of sex differences

We again conducted exploratory analyses to assess whether participants’ reported sex had any effects on perceived closeness (IOS scale) and interpersonal impressions (interpersonal *affiliation* and *experience*) of the partner avatar. Two-way ANOVAs with the factors of condition (Touch, No Touch) and sex (Female, Male) revealed no significant main effects of sex and no interactions with the experimental condition in any of these measures (see Supplementary Tables S1 and S2 for details).

## Discussion (Study 2)

The results of Study 2 replicate the results of Study 1 and demonstrate that the experience of exchanging interpersonal virtual touch with another avatar through the visual modality reduces self-reported social affiliation, including perceived closeness and impressions, towards the person behind the other avatar. Interpersonal impressions scores were lower in Study 2 than in Study 1, consistent with a shorter duration of time for participants to get to know their interaction partner, however the negative effects of touch on affiliation persisted.

Physiological measures taken during the experiment show that across both Touch and No Touch conditions, actively engaging in the virtual environment and interacting with another avatar increased skin conductance and heart rate variability, and decreased heart rate, compared to passively watching video scenes of the virtual environment and avatar interactions. Participants in the Touch condition also showed greater tonic skin conductance than those in the No Touch condition during the virtual portion of the experiment, but there was no significant difference when comparing the time period before the partner dance to the period during the dance, when virtual touch occurred.

## General discussion

In two studies we examined whether the experience of exchanging interpersonal touch with another virtual avatar during a dancing interaction, without any tactile input, would effectively function as a non-verbal signal to promote social affiliation. The results demonstrate that virtual touch impacted how participants perceived the virtual interaction, although in the opposite direction than expected. Across both studies, participants in the virtual touch condition reported significantly lower closeness towards the person behind the other avatar compared to participants in the condition where no virtual touch was exchanged. The same pattern of results was also obtained for self-reported impressions of the interpersonal experience with the virtual avatar. In Study 1, participants who exchanged virtual touch also reported significantly lower interpersonal affiliation towards the person behind the other avatar compared to those who did not exchange touch, but this effect was not significant in Study 2 when the overall duration of the interaction was shorter. The experience of virtual touch did not affect the interpersonal distance that participants maintained between their avatar and the partner avatar at the end of the interaction and also did not affect physiological arousal during the virtual touch component of the interaction.

The direction of the results was somewhat surprising as we hypothesized that experiencing virtual touch would promote rather than impede social affiliation, consistent with previous studies employing face-to-face scenarios involving touch^10–12^ and remotely-mediated touch set-ups^53,54^. However, there is evidence from studies of in-person human touch^17^ and mediated touch interactions^20,72^ demonstrating that receiving touch, especially from strangers, can cause discomfort. Studies point to the role of contextual cues from the senses other than touch that can readily modify how the tactile component of the touch is perceived^17,19–21,73^. The same touch stimulus can be felt and perceived differently and carry a different affective valence, either promoting or impeding social affiliation, depending on contextual cues from the other senses^73^. In the present study, contextual cues communicated through the visual modality provided all of the sensory information about the touch experience as no tactile sensation was available. The visualized features of the virtual social interaction, the partner avatar, and/or the virtual environment, which were novel to the participants, likely contributed to shifting the perception of the virtual touch experience towards social aversion, as seen in previous studies of mediated touch from a stranger^20,72^.

The duration of touch between avatars and areas of the body where touch occurred may have contributed to how virtual touch was experienced. Social touch can be classified according to the duration and quality of physical contact^3^. Midas touch involves a brief, intentional touch referred to as a simple touch. The physical contact represented virtually in the present study was closer to prolonged touch, which typically involves a longer duration of contact as well as the application of pressure, as in holding hands or embracing^3^. This type of touch is often used to convey affection in face-to-face interactions^74^. We chose an interaction scenario where a longer duration of physical contact would conventionally occur (i.e., partner dancing) to ensure that participants were aware of the contact taking place, as it was only represented through visual cues. However, it’s possible that this longer duration of touch, having a more affectionate connotation, led to discomfort in the interaction compared to the same scenario where no touch occurred. Furthermore, touch was exchanged across different areas of the body of the avatars, including the hands, torso, and upper legs. Areas of the body such as the hands and arms are reported as more appropriate to receive a touch from a stranger than the torso^7^. The more intimate form of touch used here may have contributed to the experience being perceived as aversive compared to studies that employ a touch to the arm, shoulder, or hand, as seen in typical Midas touch scenarios that promote social affiliation towards a stranger^10–13,15^.

The present results demonstrate that participants reliably responded to the virtual touch manipulation, showing that the representation of virtual touch through the visual modality can impact one’s impressions about the experience and their interaction partner. A similar study that examined the influence of prosocial gestures between robot-like avatars showed that virtual touch, without tactile input, also affected how the participants evaluated the interaction^35^. Specifically, exchanging prosocial gestures that included touch (high fives or hugs) with a fellow robot avatar within a video-game environment increased self-reported relatedness to the other player compared to not exchanging any gestures or receiving negative gestures (teasing or laughter)^35^. Although the direction of findings in this study are opposite to ours, the contextual differences between the two studies, both in the nature of the interactions (e.g., brief vs. long duration) and the types of avatars used (i.e., depictions of robots vs. humans), likely contributed to these opposite effects. For instance, embodying a human compared to a robot avatar has been shown to lead to a higher sense of body ownership, greater connection to reality, and less risky behaviors in a virtual environment^75^. Nevertheless, both the findings of ^35^ and our own demonstrate that simulating the tactile component of touch in a virtual setting is not necessary to convey social signals communicated by the act of touching; the visualized physical contact cues themselves can direct how the social experience is perceived. Taken together, the findings of the present study alongside previous work suggest that, as with in-person touch, the influence of virtual touch on how a social interaction is perceived depends on the interaction context.

In studies of in-person touch, body language and vocal cues, such as facial expressions and tone of voice, may contribute to unconscious bias on the part of the confederate when not blinded. However, the experimenter controlling the confederate avatar in the present studies had no in-person interactions with the participants. In addition, all visual features of avatar interactions were automated and there were no voice exchanges between avatars. Given these features of the experiment, it’s highly unlikely that unconscious cues were a confounding factor in the present studies. In fact, virtual set-ups have been proposed as an effective solution to avoiding confederate bias in studying the social influence of touch^53^.

There was no evidence in the present study that virtual touch affected social behaviour in the virtual environment. Studies have previously shown that interpersonal distance between individuals can be used as an indicator of social affiliation^58,60^ and that interpersonal distance influences how avatars position themselves in relation to other avatars in virtual environments^36,39,40^. However, the virtual touch manipulation used here did not have an influence on how close participants positioned their avatar to sit beside the confederate avatar. In real-world interactions, interpersonal distance involves multi-sensory signaling of social information, including proximity as well as body posture, eye gaze, and potentially smell. It is possible that the representation of interpersonal distance in our virtual set-up was not a strong enough social cue.

Consistent with previous studies^76,77^, participants had different physiological responses when exploring the virtual environment and/or interacting with another avatar compared to passively watching a video of the same environment. Participants from both Touch and No Touch conditions had higher skin conductance responses during the virtual component of the experiment compared to video watching. Both groups also showed a significant decrease in heart rate and a significant increase in heart rate variability during the virtual component. However, there was little evidence that dancing in the virtual environment affected physiological responses when compared to virtual activities prior to dancing. A significant increase in skin conductance responses was observed during the Dance event compared to the Pre-Dance event, but only when the video and virtual components of the experiment were considered together (i.e., main effect of event). Participants in the Touch condition also had significantly higher tonic skin conductance compared to those in the No Touch condition, although this pattern was observed both prior to and during the virtual dance. Overall, there was no clear evidence to indicate that the experience of virtual touch had a measurable effect on physiological responding compared to other virtual activities. A previous study of the physiological responses to virtual touch showed that viewing touch on one’s virtual body in first-person produces stronger skin conductance responses than viewing the same stimulus in third-person^51^. The present studies employed the third-person view, which may have led to less robust skin conductance responses to virtual touch.

The visual representation of virtual touch employed in the present studies inherently involved a depiction of physical proximity between avatars. There is evidence that the processing of interpersonal touch and interpersonal distance cues is related. For instance, when visual input is maintained but haptic input is switching from objectual to interpersonal touch, faster responses are provided in body-related mental representation tasks, as if targets are perceived at a closer distance to one’s own body^78^. Comfort with interpersonal distance and social touch are also associated^79^, and both are influenced by adverse life experience and linked by underlying neural processing^80^. Furthermore, mental disorders that are characterized by increased social distancing, such as depression^81^, can affect neural processing of social touch stimuli^82^. This body of evidence indicates that the role of social distance cues in the visual representation of social touch, as well as individual preferences regarding social proximity and comfort with touch^83^, are important to consider in future investigations of virtual touch interactions.

The response to social touch can be influenced by other interpersonal factors, such as one’s familiarity and relationship with the toucher^7,20,72^, one’s cultural background^84–86^, as well as the gender and/or sex of the toucher and the person being touched^8,19,84^. In the present studies, we asked participants about their sex to standardize pairing with an opposite sex avatar. Although our pre-planned analyses were not powered to examine the contribution of sex to the main outcomes of interest, exploratory analyses revealed that participants’ sex did not significantly influence any of the social affiliation measures and did not interact with the touch condition. As we only examined the exchange of virtual touch between opposite-sex participants and avatars, further investigation of the influence of participant and avatar sex, as well as gender identity and sexual orientation is needed. There is evidence to indicate that these factors have an influence on how social touch and virtual touch are perceived^52,87,88^, with the virtual context adding a further level of complexity where the sex of participants’ and their avatars’ bodies can be made to differ^87^.

Given the relevance of touch in social interactions and the development of increasingly complex and immersive virtual spaces, such as the metaverse, understanding how virtual touch is experienced in these contexts requires greater consideration^25,89^. The important role of traditional forms of touch in wellbeing^83^ and its stress-buffering effects are well documented^90,91^. A growing body of work also demonstrates that experiences in virtual environments can impact the offline self with respect to self-perception, well-being, and health behaviors^41,92–94^. An intriguing possibility is that virtual touch interactions may also impact aspects of health and well-being offline. These impacts may be both positive or negative, depending on how the context and how the virtual touch interaction is perceived. For instance, sharing virtual touch with a familiar individual may bring comfort at a distance, as previously demonstrated in haptic exchanges^20,95^. On the other hand, being exposed to inappropriate touching in virtual reality environments, as reported in some existing metaverse settings^96^, may lead to real-world mental health impacts. As shown in the present study, virtual touch has real social implications and the parameters of its influence require more thorough investigation.

There are several limitations to consider when interpreting the results of the present studies. The participant sample consisted of undergraduate students, which limits the generalizability of the findings to a younger population of adults who are generally accustomed to social interactions mediated via digital technologies. As mentioned above, the exchange of virtual touch employed here was limited to opposite-sex avatar dyads. Also, the influence of participant sex was only examined in an exploratory fashion and the contribution of gender and sexual orientation was not considered. Finally, the studies were conducted within one type of virtual setting. Further examination of the social implications of virtual touch in other settings, such as virtual reality environments, should include a larger and more diverse sample population.

## Conclusion

The findings of the present study demonstrate that exchanging virtual touch between human-like avatars lead to lower social affiliation toward the interaction partner. Importantly, virtual touch was only represented through visual cues indicating that this modality is sufficient to communicate interpersonal touch signals that impact social affiliation. These findings indicate that the social communication function of touch extends to virtual simulations without physical feedback and have implications for understanding how intimate interpersonal exchanges impact social experiences in virtual environments such as the metaverse. Future studies should extend these findings to a larger and more diverse sample and investigate how different typologies of virtual social touch alongside individual differences may contribute to the impact of the virtual touch experience.

### Supplementary Information

Supplementary Tables.

Supplementary Legends.

Supplementary Video S1.

Supplementary Video S2.

## Data Availability

The datasets generated and analysed during the current study are available from the corresponding author on reasonable request.
