# Supplementary material for: Virtually simulated interpersonal touch negatively affects perceived closeness and social affiliation to an avatar partner
Source: Sci Rep. 2024 Jan 16;14:1347. doi: 10.1038/s41598-024-51773-6 (PMC10791684; doi:10.1038/s41598-024-51773-6)
Supplement: Supplementary file 1 — Supplementary Tables. [file 41598_2024_51773_MOESM1_ESM.docx]

**Supplementary Tables**

**Table S1.** Descriptive statistics of self-report measures with the variables of condition (Touch, No Touch) and participant sex included.

|  |  | **Female** | | | | | | |  | **Male** | | | | | | |
| --- | --- | --- | --- | --- | --- | --- | --- | --- | --- | --- | --- | --- | --- | --- | --- | --- |
|  |  | **Touch** | | |  | **No Touch** | | |  | **Touch** | | |  | **No Touch** | | |
| **Measure** | **Study** | **n** | **Mean** | **SD** |  | **n** | **Mean** | **SD** |  | **n** | **Mean** | **SD** |  | **n** | **Mean** | **SD** |
| Inclusion of Other in the Self (IOS) | 1 | 21 | 94.9 | 79.3 |  | 19 | 179.0 | 54.0 |  | 11 | 152.1 | 84.4 |  | 9 | 162.2 | 84.2 |
|  | 2 | 11 | 121.8 | 98.3 |  | 11 | 176.3 | 86.7 |  | 8 | 79.1 | 88.8 |  | 8 | 167.1 | 54.7 |
| Interpersonal Experience | 1 | 21 | 4.3 | 1.7 |  | 19 | 5.3 | 0.9 |  | 11 | 4.8 | 1.3 |  | 10 | 5.2 | 1.0 |
|  | 2 | 11 | 3.9 | 1.2 |  | 11 | 4.5 | 0.9 |  | 8 | 2.9 | 1.5 |  | 8 | 4.7 | 1.2 |
| Interpersonal Affiliation | 1 | 21 | 3.8 | 1.5 |  | 19 | 4.7 | 1.0 |  | 11 | 4.4 | 1.3 |  | 10 | 4.9 | 0.8 |
|  | 2 | 11 | 3.5 | 1.4 |  | 11 | 4.1 | 0.7 |  | 8 | 3.6 | 1.6 |  | 8 | 4.0 | 1.0 |

**Table S2.** Results of two-way ANOVA analyses of self-report measures with the variables of condition (Touch, No Touch) and participant sex (Female, Male) included.

| **Measure** | **Study** | **Variable** | **df** | **F** | ***p*** | ***η_p_^2^*** |
| --- | --- | --- | --- | --- | --- | --- |
| Inclusion of Other in the Self (IOS) | 1 | Condition | 1, 56 | 5.38 | 0.024* | 0.09 |
|  |  | Sex | 1, 56 | 0.99 | 0.324 | 0.02 |
|  |  | Condition X Sex Interaction | 1, 56 | 3.31 | 0.074 | 0.06 |
|  | 2 | Condition | 1, 34 | 6.44 | 0.016* | 0.16 |
|  |  | Sex | 1, 34 | 0.85 | 0.362 | 0.02 |
|  |  | Condition X Sex Interaction | 1, 34 | 0.36 | 0.554 | 0.01 |
| Interpersonal Experience | 1 | Condition | 1, 57 | 3.60 | 0.063 | 0.06 |
|  |  | Sex | 1, 57 | 0.42 | 0.522 | 0.01 |
|  |  | Condition X Sex Interaction | 1, 57 | 0.63 | 0.429 | 0.01 |
|  | 2 | Condition | 1, 34 | 8.96 | 0.005* | 0.21 |
|  |  | Sex | 1, 34 | 1.03 | 0.317 | 0.03 |
|  |  | Condition X Sex Interaction | 1, 34 | 2.84 | 0.101 | 0.08 |
| Interpersonal Affiliation | 1 | Condition | 1, 57 | 4.63 | 0.036* | 0.08 |
|  |  | Sex | 1, 57 | 2.07 | 0.155 | 0.04 |
|  |  | Condition X Sex Interaction | 1, 57 | 0.34 | 0.565 | 0.01 |
|  | 2 | Condition | 1, 34 | 1.43 | 0.239 | 0.04 |
|  |  | Sex | 1, 34 | 0.00 | 0.951 | 0.00 |
|  |  | Condition X Sex Interaction | 1, 34 | 0.03 | 0.863 | 0.00 |

*p < 0.05

**Table S3.** Results of a repeated measures three-way ANOVA analysis of absolute skin conductance level (SCL) values in Study 1, Study 2, and both studies combined.

| **Study** | **Variable** | **df** | **F** | ***p*** | ***η_p_^2^*** |
| --- | --- | --- | --- | --- | --- |
| Study 1 | Event | 1, 52 | 0.55 | 0.460 | 0.01 |
| (N = 54) | Media | 1, 52 | 6.44 | 0.014* | 0.11 |
|  | Condition | 1, 52 | 3.06 | 0.086 | 0.06 |
|  | Event x Media | 1, 52 | 0.00 | 0.991 | 0.00 |
|  | Event x Condition | 1, 52 | 1.54 | 0.220 | 0.03 |
|  | Media x Condition | 1, 52 | 2.29 | 0.136 | 0.04 |
|  | Event x Media x Condition | 1, 52 | 0.54 | 0.466 | 0.01 |
| Study 2 | Event | 1, 29 | 3.70 | 0.064 | 0.11 |
| (N = 31) | Media | 1, 29 | 2.11 | 0.158 | 0.07 |
|  | Condition | 1, 29 | 1.65 | 0.209 | 0.05 |
|  | Event x Media | 1, 29 | 8.20 | 0.008** | 0.22 |
|  | Event x Condition | 1, 29 | 1.98 | 0.170 | 0.06 |
|  | Media x Condition | 1, 29 | 1.85 | 0.185 | 0.06 |
|  | Event x Media x Condition | 1, 29 | 0.91 | 0.347 | 0.03 |
| Both Studies | Event | 1, 83 | 3.68 | 0.059 | 0.04 |
| (N = 85) | Media | 1, 83 | 8.75 | 0.004** | 0.10 |
|  | Condition | 1, 83 | 4.94 | 0.029* | 0.06 |
|  | Event x Media | 1, 83 | 2.74 | 0.102 | 0.03 |
|  | Event x Condition | 1, 83 | 2.77 | 0.100 | 0.03 |
|  | Media x Condition | 1, 83 | 4.36 | 0.040* | 0.05 |
|  | Event x Media x Condition | 1, 83 | 0.04 | 0.847 | 0.00 |

*p < 0.05, **p < 0.01

**Table S4.** Results of a repeated measures three-way ANOVA analysis of the frequency of skin conductance responses (SCR) in Study 1, Study 2, and both studies combined.

| **Study** | **Variable** | **df** | **F** | ***p*** | ***η_p_^2^*** |
| --- | --- | --- | --- | --- | --- |
| Study 1 | Event | 1, 52 | 2.36 | 0.130 | 0.04 |
| (N = 54) | Media | 1, 52 | 9.02 | 0.004** | 0.15 |
|  | Condition | 1, 52 | 0.96 | 0.332 | 0.02 |
|  | Event x Media | 1, 52 | 14.94 | 0.0003*** | 0.22 |
|  | Event x Condition | 1, 52 | 0.12 | 0.736 | 0.00 |
|  | Media x Condition | 1, 52 | 0.85 | 0.361 | 0.02 |
|  | Event x Media x Condition | 1, 52 | 1.06 | 0.307 | 0.02 |
| Study 2 | Event | 1, 29 | 4.55 | 0.042* | 0.14 |
| (N = 31) | Media | 1, 29 | 8.57 | 0.007** | 0.23 |
|  | Condition | 1, 29 | 1.30 | 0.263 | 0.04 |
|  | Event x Media | 1, 29 | 2.53 | 0.123 | 0.08 |
|  | Event x Condition | 1, 29 | 0.22 | 0.641 | 0.01 |
|  | Media x Condition | 1, 29 | 1.12 | 0.298 | 0.04 |
|  | Event x Media x Condition | 1, 29 | 0.03 | 0.872 | 0.00 |
| Both Studies | Event | 1, 83 | 6.69 | 0.011* | 0.07 |
| (N = 85) | Media | 1, 83 | 15.19 | 0.0002*** | 0.15 |
|  | Condition | 1, 83 | 0.00 | 0.955 | 0.00 |
|  | Event x Media | 1, 83 | 3.52 | 0.064 | 0.04 |
|  | Event x Condition | 1, 83 | 0.22 | 0.639 | 0.00 |
|  | Media x Condition | 1, 83 | 1.75 | 0.190 | 0.02 |
|  | Event x Media x Condition | 1, 83 | 1.37 | 0.245 | 0.02 |

*p < 0.05, **p < 0.01, ***p < 0.001

**Table S5.** Results of a repeated measures three-way ANOVA analysis of the average heart beats per minute (HR) in Study 1, Study 2, and both studies combined.

| **Study** | **Variable** | **df** | **F** | ***p*** | ***η_p_^2^*** |
| --- | --- | --- | --- | --- | --- |
| Study 1 | Event | 1, 25 | 2.21 | 0.150 | 0.08 |
| (N = 27) | Media | 1, 25 | 14.90 | 0.0007*** | 0.37 |
|  | Condition | 1, 25 | 0.11 | 0.741 | 0.00 |
|  | Event x Media | 1, 25 | 1.15 | 0.295 | 0.04 |
|  | Event x Condition | 1, 25 | 0.03 | 0.859 | 0.00 |
|  | Media x Condition | 1, 25 | 0.24 | 0.626 | 0.01 |
|  | Event x Media x Condition | 1, 25 | 0.64 | 0.432 | 0.02 |
| Study 2 | Event | 1, 13 | 0.40 | 0.539 | 0.03 |
| (N = 15) | Media | 1, 13 | 11.04 | 0.006** | 0.46 |
|  | Condition | 1, 13 | 0.00 | 0.995 | 0.00 |
|  | Event x Media | 1, 13 | 0.03 | 0.860 | 0.00 |
|  | Event x Condition | 1, 13 | 2.69 | 0.125 | 0.17 |
|  | Media x Condition | 1, 13 | 0.87 | 0.368 | 0.06 |
|  | Event x Media x Condition | 1, 13 | 0.33 | 0.577 | 0.02 |
| Both Studies | Event | 1, 40 | 0.22 | 0.638 | 0.01 |
| (N = 42) | Media | 1, 40 | 26.50 | 0.0001*** | 0.40 |
|  | Condition | 1, 40 | 0.10 | 0.755 | 0.00 |
|  | Event x Media | 1, 40 | 0.62 | 0.434 | 0.02 |
|  | Event x Condition | 1, 40 | 1.72 | 0.197 | 0.04 |
|  | Media x Condition | 1, 40 | 0.95 | 0.336 | 0.02 |
|  | Event x Media x Condition | 1, 40 | 0.82 | 0.369 | 0.02 |

**p < 0.01, ***p < 0.001

**Table S6.** Results of a repeated measures three-way ANOVA analysis of the standard deviation of interbeat intervals (SDNN) in Study 1, Study 2, and both studies combined.

| **Study** | **Variable** | **df** | **F** | ***p*** | ***η_p_^2^*** |
| --- | --- | --- | --- | --- | --- |
| Study 1 | Event | 1, 25 | 0.18 | 0.676 | 0.01 |
| (N = 27) | Media | 1, 25 | 14.61 | 0.0008* | 0.37 |
|  | Condition | 1, 25 | 0.02 | 0.890 | 0.00 |
|  | Event x Media | 1, 25 | 1.68 | 0.207 | 0.06 |
|  | Event x Condition | 1, 25 | 0.10 | 0.760 | 0.00 |
|  | Media x Condition | 1, 25 | 0.03 | 0.866 | 0.00 |
|  | Event x Media x Condition | 1, 25 | 1.82 | 0.189 | 0.07 |
| Study 2 | Event | 1, 13 | 1.50 | 0.242 | 0.10 |
| (N = 15) | Media | 1, 13 | 3.74 | 0.075 | 0.22 |
|  | Condition | 1, 13 | 0.03 | 0.859 | 0.00 |
|  | Event x Media | 1, 13 | 2.50 | 0.138 | 0.16 |
|  | Event x Condition | 1, 13 | 0.00 | 0.996 | 0.00 |
|  | Media x Condition | 1, 13 | 2.75 | 0.122 | 0.17 |
|  | Event x Media x Condition | 1, 13 | 0.00 | 0.962 | 0.00 |
| Both Studies | Event | 1, 40 | 0.10 | 0.753 | 0.00 |
| (N = 42) | Media | 1, 40 | 16.38 | 0.0002* | 0.29 |
|  | Condition | 1, 40 | 0.07 | 0.793 | 0.00 |
|  | Event x Media | 1, 40 | 0.02 | 0.881 | 0.00 |
|  | Event x Condition | 1, 40 | 0.00 | 0.955 | 0.00 |
|  | Media x Condition | 1, 40 | 0.70 | 0.407 | 0.02 |
|  | Event x Media x Condition | 1, 40 | 0.67 | 0.418 | 0.02 |
