# Supplementary material for: Virtually simulated interpersonal touch negatively affects perceived closeness and social affiliation to an avatar partner
Source: Sci Rep. 2024 Jan 16;14:1347. doi: 10.1038/s41598-024-51773-6 (PMC10791684; doi:10.1038/s41598-024-51773-6)
Supplement: Supplementary file 2 — Supplementary Legends. [file 41598_2024_51773_MOESM2_ESM.docx]

**Supplementary Video Captions**

**Supplementary Video S1. Representative video of the three styles of dance animations in the Touch condition.** A similar 2 min video of the same avatars dancing was shown to participants during the introductory portion of the experiment (Touch - Video Dance event). The DJ avatar present in the background was only included in Study 2.

**Supplementary Video S2. Representative video of the three styles of dance animations in the No Touch condition.** A similar 2 min video of the same avatars dancing was shown to participants during the introductory portion of the experiment (No Touch - Video Dance event).
